# Development and Field Tests of a Deep-Sea Laser-Induced Breakdown Spectroscopy (LIBS) System for Solid Sample Analysis in Seawater

**DOI:** 10.3390/s20247341

**Published:** 2020-12-21

**Authors:** Chunhao Liu, Jinjia Guo, Ye Tian, Chao Zhang, Kai Cheng, Wangquan Ye, Ronger Zheng

**Affiliations:** College of Information Science & Engineering, Ocean University of China, Qingdao 266100, China; liuchunhao@stu.ouc.edu.cn (C.L.); ytian@ouc.edu.cn (Y.T.); zhangc_ouc@163.com (C.Z.); chengkai@ouc.edu.cn (K.C.); yewangquan@ouc.edu.cn (W.Y.); rzheng@ouc.edu.cn (R.Z.)

**Keywords:** LIBS instrumentation, underwater solid analysis, field test, in-situ detection, deep-sea

## Abstract

In recent years, the investigation and exploitation of hydrothermal region and polymetallic mineral areas has become a hot topic. The emergence of underwater vehicle platforms has made it possible for new chemical sensors to be applied in marine in-situ detection. Laser-induced breakdown spectroscopy (LIBS), with its advantages of rapid real-time analysis, sampling without pretreatment, simultaneous multi-element detection and stand-off detection, has great potential in marine applications. In this paper, a newly more compact and lighter underwater LIBS system based on the LIBSea system named LIBSea II was developed and tested both in the laboratory and sea trials. The system consists of a Nd:YAG single-pulse laser at 1064 nm, a fiber spectrometer, optical layout, a power supply module and an internal environment sensor. The system is encapsulated in a pressure vessel (Φ 190 mm × L 588 mm) with an optical window on the end cap. Experimental parameters of the system including laser energy and delay time were firstly optimized in the laboratory. Then, field test of the system in nearshore was performed with various samples, including pure metal and alloy samples as well as a manganese nodule sample from deep sea, to verify the detection performance of the LIBSea II system. In 2019, the system was deployed on a remotely operated vehicle (ROV) of Haima for deep sea trial, and atomic lines of K, Na, Ca and strong molecular bands of CaOH from a carbonate rock sample were obtained for the first time at depths of 1400 m. These results show that the LIBSea II system has great potential to be used in deep-sea geological exploration.

## 1. Introduction

Deep-sea hydrothermal area and polymetallic mineral areas are widely distributed all over the world. Due to their abundance of metal resources, these areas have become the focus of marine research in recent years. Traditional analytical methods are diversified, and it is usually required to analyze the samples after obtaining them from the deep sea. The number of samples that can be retrieved limits the range and spatial resolution of the information obtained, and the information is not immediately available for feedback since the analysis is typically performed in a laboratory [1]. There are not many analytical techniques available for the in-situ exploration of underwater sites, and therefore the development of in-situ instruments is highly demanded, in particular those based on optical technologies. Oceanic in-situ sensors based on spectroscopic technology have many advantages, such as simultaneous detection of multiple components, non-contact, high speed in analysis, wide adaptability, strong flexibility, and are becoming one of the hot topics in marine research. Recently, with the rapid development of underwater vehicle platforms, it will be convenient that kinds of in-situ optical sensors are used for ocean observation and exploration. 

As a newly developed spectroscopic technology, laser-induced breakdown spectroscopy (LIBS) has been widely applied in various fields [2,3,4,5,6], especially the ChemCam system applied in Mars exploration has attracted the worldwide attention [7,8,9]. However, due to the high density, compression resistance and high heat conductivity of water [10], compared with the successful applications in air environment, it is still a big challenge to apply LIBS technology to deep-sea research. Up to now, most of the underwater LIBS studies are carried out in the laboratory, including the laser irradiation method [11,12,13], spectrum detection mode [14] and underwater high-pressure effect [15,16,17,18] and data processing of underwater LIBS spectrum [19,20]. Only a few scientific research institutions have carried out the in-situ oceanic applications of LIBS technology.

The University of Malaga in Spain has reported two sets of equipment that can be used for archaeological investigations in shallow sea [21,22]. Both of them consist of a ship-borne part and a probe part, which are connected by an optical fiber for transmission of the ablation laser and the LIBS signal. Compared with the first-generation equipment, the second-generation equipment named AQUALAS 2.0 reduces the weight of the ship-borne part (from 300 kg to 150 kg) and with a longer optical fiber. The use of multi-pulse laser detection in the second-generation system has greatly improved the performance of the instrument. Recently, the University of Tokyo and Ocean University of China have applied LIBS technology to in-situ detection in deep sea. The University of Tokyo developed the 3000 m rated I-SEA system and ChemiCam system successively [1,23,24]. They all adopt a segmented design which is divided into two parts: the main housing and the fiber probe head. The two parts are connected by a fiber optic cable. When working, the liquid detection was carried out in the main housing, while the solid detection was completed by the optical fiber probe with the help of the remotely operated vehicle (ROV) manipulator. The I-SEA system has performed multi-elements analysis in seawater as well as phosphatised carbonate samples at a depth of 200 m [23,24]. Later, the ChemiCam system has measured the hydrothermal fluid and sediment successfully at the Iheya North field in the Okinawa trough at a depth over 1000 m [24], and the in-situ quantitative analysis of deep-sea minerals in the hydrothermal area was carried out for the first time [25]. Ocean University of China developed a compact 4000 m rated deep-sea LIBS system (LIBSea) by using the integrated design in 2015. The system has performed the detection of hydrothermal fluid in Manus area and the measurement of seawater profile with the diving of Faxian ROV [26,27]. The major metallic elements, Na, Ca, K, Mg, as well as Li, were clearly detectable in the spectra obtained at sea floor in the hydrothermal area, and the elemental profiles of LIBS signals of K and Ca were also obtained during the sea trials.

In order to realize the underwater LIBS system that can be easily gripped by the ROV manipulator, a more compact, lighter and lower power consumption deep-sea LIBS system (LIBSea II) is developed on the basis of the LIBSea system. When working, the system can be directly gripped by the ROV manipulators with the functioning of T-handles for the detection of submerged solid targets. In this paper, the configurations and specifications of LIBSea II system will be first introduced in detail. Then the optimization of system parameters in the laboratory together with the comparative tests of metal targets in the shallow sea will be shown. Field-test results including the quantitative analysis of alloy targets and manganese nodule from deep sea in shallow sea and the LIBSea II system with carbonate rock sample deployed on remotely operated vehicles for deep sea detection will also be presented.

## 2. Experimental Setup

**Figure 1:**
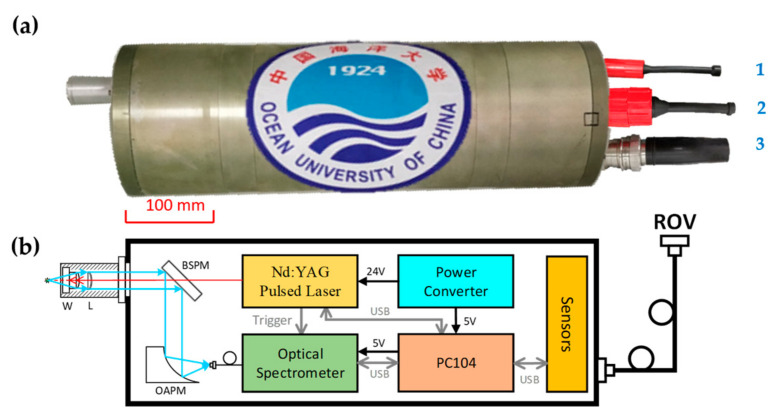
(**a**) The photo of the newly developed underwater LIBS device (LIBSea II). (**b**) Schematic of the LIBSea II system showing the optical layout, pulsed laser, optical spectrometer, embedded computer, power converter and environment sensor.

Figure 1a is the newly developed underwater system named LIBSea II. It is packed into one cylindrical pressure housing made of 7075 aluminum alloy which was treated by surface oxidation blackening to retard corrosion. The size of the system is 588 mm in length and 190 mm in diameter capable of withstanding 50 MPa with a 25% safety margin. The total weight of the LIBSea II system is 28 kg in air, which is much lighter than that of the LIBSea system (56 kg in air) [26]. Because the system weighs only 12 kg in water, it can be easily hold by a manipulator of ROV. The system is usually deployed with ROVs for deep-sea measurements. The ROV is a powerful underwater vehicle for subsea exploration, attached to a photoelectric composite umbilical cable to the deck over which the communication signal and the electric power are transmitted. With the powerful communication capabilities of the ROV based on its umbilical cable, the entire system could operate in interactive mode through high-rate interfaces (Gigabit Ethernet interface). Interactive mode enables operators on the deck to communicate with underwater instruments through remote desktop and the returned spectrum data can be displayed on the computer screen in real time and will help us analyze the quality of the data in time. It can be seen in Figure 1a that there are three connecting ports on the end of cylindrical housing which can be adapted to most ROVs in China The first port is used to connect the low-rate interfaces on ROVs for automatic mode or laboratory tests, and another two for connecting the high-rate interfaces on different ROVs to display data in real time at the deck terminal or the internal communication between other systems on requiring. During the experiment of LIBSea II in deep sea, the power supply of the system is provided by the ROV the power and ethernet cables. A power converter in the pressure housing is used to realize the conversion of DC to DC. The output voltages are 24 V DC and 5 V DC that were supplied to the Nd:YAG pulsed laser and the embedded computer, respectively. The power supply for the optical spectrometer is provided by the embedded computer USB interface. When the ROV starts to supply power to the system, the embedded computer in the pressure housing will start automatically, and the operator controls the LIBSea II system through the remote desktop. And an integrated USB environment sensor is used to monitor the temperature, humidity and pressure to monitor the internal environment of the system and ensure the safety during the experiments in deep sea. There is a specially designed optical probe on the front end of the housing for submerged solids detection.

To make the system compact in size and light in weight, the core instruments were carefully selected and modified to meet the requirements of underwater working. The LIBSea II system is shown schematically in Figure 1b. The cross-sections of the cylindrical pressure housings are plotted in solid line giving a reference space measure for the whole package of the spectral systems. A Q-switched neodymium-doped yttrium aluminum garnet (Nd:YAG) laser (M-NANO, Montfort, Götzis, Austria) operating in a fundamental frequency with a pulse duration of 10 ns was used as the excitation source giving a maximum pulse energy of 40 mJ at the repetition rate of 2–25 Hz. The laser can be operated from 0 to 50 degrees Celsius for adapting to the lower temperature of deep-sea environment. Also, the core instrument can be cooled by thermal conduction through the walls of the high-pressure housing. Although the laser wavelength of 1064 nm is absorbed seriously in water, it can be used as the excitation source for a better detection capability [28]. The whole laser package was modified into a compact size of 258 mm (L) × 78 mm (W) × 64 mm (H) for underwater operation. To obtain a high-quality LIBS signal, special attentions are paid on the design of the focusing lens of the LIBSea II system. Single plano-convex lens is not suitable to be used as the focusing lens in underwater LIBS measurement, because of its large spherical aberration, which can lead to a low repeatability of the underwater LIBS signals [29]. There are many ways to reduce the spherical aberration, such as using the doublet lens or the combination of two single lens. As shown in our previous work [29,30], the application of a doublet lens together with a large focusing angle can greatly improve the stability of the plasma and lead to high-quality LIBS signals. However, in the practical case, there is a certain safety risk because the doublet lens may be damaged by the high-energy laser pulse. Therefore, in this work, the combination of double plano-convex lenses has been used in LIBSea II system. We have verified in the laboratory that the spherical aberration can be clearly reduced with this focusing arrangement and the quality of LIBS signals can be improved [31]. The optical probe consists of a specially designed lens (W, shown in Figure 1b, is a specially designed fused silica plano-convex lens with a 11.82 mm curvature radius and 16.36 mm thickness) and a fused silica plano-convex lens (L, shown in Figure 1b) with 50.2 mm in focal length. At the same time, the specially designed lens is also served as a pressure-resistant optical window. It also has to pass the hydrostatic pressure test of 62.5 MPa for deep-sea experiments. The plasma emission of seawater is induced by the tightly focused laser beam at a 7.8 mm distance away which can avoid the strong laser absorption in water and ensure more lights throughput of plasma signal from the optical window. The emission is then collected by the same optics. After being reflected by a back side polished mirror (BSPM, Reflectance > 95% at 400–800 nm and Transmission > 90% at 1064 nm) and an off-axis parabolic mirror (OAPM, focusing a collimated beam without chromatic aberrations), the collected LIBS signal is then coupled into a compact optical spectrometer (AvaSpec-Mini2048, Avantes, Apeldoorn, Netherlands), which offers a wavelength coverage of 260–800 nm and a spectral resolution of 0.63 nm with a 2048 pixels CCD. The spectrometer was triggered by the customized pulsed laser. In order to eliminate the original delay time (1.28 μs) of the optical spectrometer, the laser can set appropriate Q-switch trigger signal to offset the delay time. The detailed specifications of LIBSea II system are listed in Table 1.

## 3. Results and Discussion

### 3.1. Optimization of System Parameters in Laboratory

**Figure 2:**
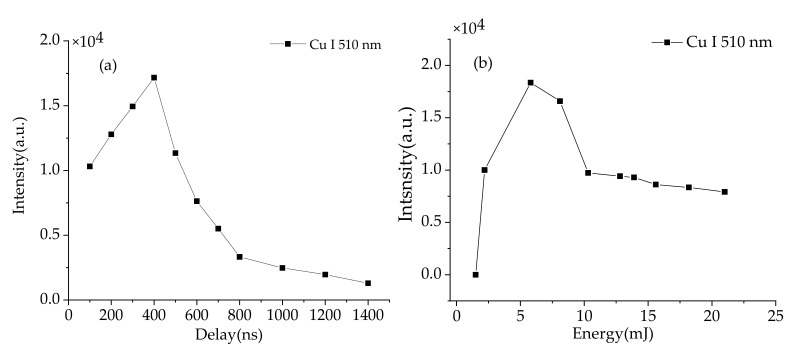
(**a**) Spectral intensity of Cu I 510 nm under different delay time. (**b**) Spectral intensity of Cu I 510 nm under different laser energy.

Experimental parameters of the system including delay time and laser energy were first optimized with a submerged copper target in laboratory. One important parameter is the detection delay time, which is used to avoid the interference of the continuum emission from the early stage plasma after the laser pulse. Figure 2a shows the peak intensity of LIBS signal at Cu I 510 nm with different delay time. It can be seen that the signal intensity tends to increase first and then decrease. Due to the strong bremsstrahlung in the early stage of plasma evolution, the continuous background of the spectral line is high. As time evolves, the plasma in water cools down rapidly and the continuous background decreases significantly. The highest peak intensity is found at a delay time of 400 ns. After this time the signal gradually weakens and disappears at 1400 ns. In addition, the optimization of laser energy is also very important for obtaining high-quality LIBS signal, especially for the underwater measurements. With low laser energy, a 100% breakdown probability cannot be achieved, while with high laser energy, the plasma in water suffers a strong shielding effect that degrades the LIBS signals. Figure 2b shows the spectral intensity of LIBS signal at Cu I 510 nm as a function of laser energy. The spectra were recorded at a delay time of 400 ns and with an average of 50 laser shots. It can be seen that as the laser energy increases, the signal intensity rises from 1 to 6 mJ. This is because when the energy is low, the laser fluence cannot reach the breakdown threshold to achieve an efficient ablation on the target surface. As the laser energy increases, the ablation mass of the target will be increased, so that the LIBS signal is enhanced. The signal intensity reaches the maximum value at 6 mJ. And then the signal began to show a clearly decrease trend with the increased energy. This is mainly because the continual increase in energy will lead to the probability of breakdown in the water medium instead of on the target surface. A strong plasma shielding phenomenon occurs in this case which leads to a clearly decrease of the LIBS signal. Based on these results, an optimal laser energy of 6 mJ and delay time of 400 ns were used for the following field tests.

### 3.2. Shallow Sea Experiments

**Figure 3:**
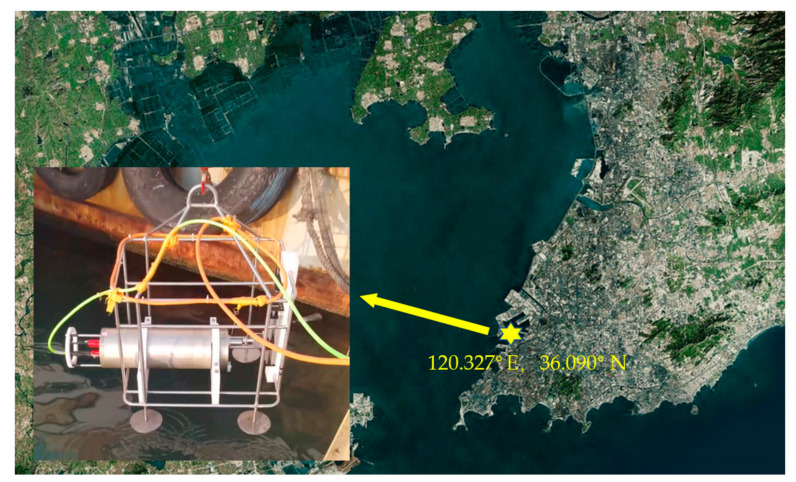
Map showing the location of nearshore underwater measurement in the Jiaozhou Bay. The system is placed on the mounting frame and lifted underwater by the shipboard motor.

In order to evaluate the performance of LIBSea II for submerged solid detection, a series of shallow-sea experiments were performed in nearshore of the Jiaozhou Bay. The system was running during 9:00 a.m. to 18:14 p.m. on 23 November 2018. The air temperature was reported from 8 °C to 17 °C and the experiment positions were exposed to a level 3 southwest wind. The measurement location is 36 °04′26″ N, 120°18′43″ E and the map of the location was shown in Figure 3. The rack-mounted system was deployed at a depth of 5 m on the seafloor for the shallow-sea test. The used samples are pure metal targets of Zn, Cu, Al, Fe, a series of Zn-Cu alloy targets with different Zn/Cu mass ratios and manganese nodules sample from deep sea. The spectra were recorded with the same experimental parameters optimized in the laboratory (i.e., 6 mJ laser energy, 400 ns delay time).

**Figure 4:**
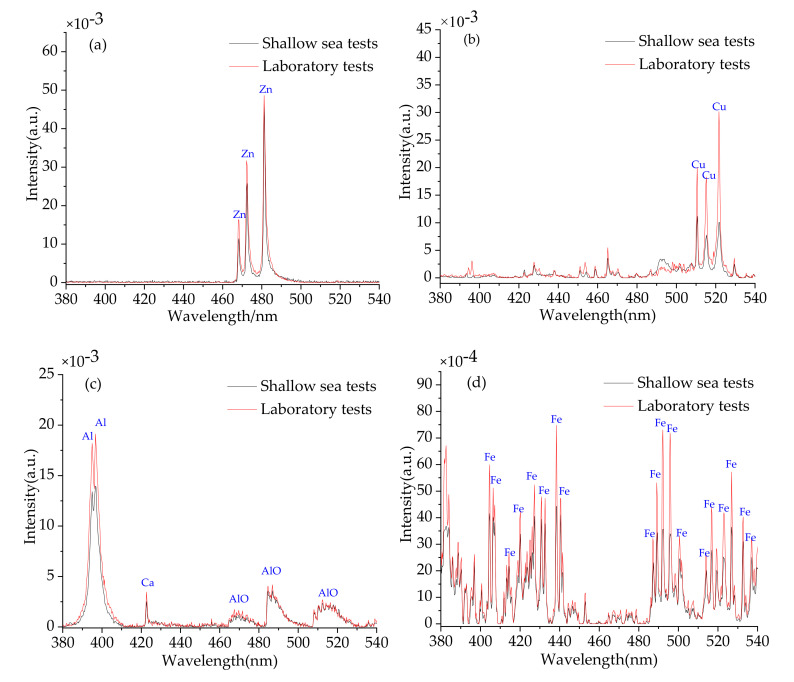
Spectra of pure metal targets of (**a**) Zn, (**b**) Cu, (**c**) Al, (**d**) Fe measured in shallow sea (black line) and in laboratory (red line) for comparison.

Figure 4a–d show the spectra of pure metal targets of Zn, Cu, Al, and Fe measured by LIBSea II system in shallow sea (black line) as well as in laboratory (red line). 

The spectra were preprocessed by background subtraction and normalization for comparison purpose due to the different water environment. The background was subtracted using symmetrically reweighted penalized least squares method [32], and each spectrum was normalized by the total intensity of the whole spectrum [1,13]. According to the NIST atomic spectra database, the characteristic lines of the corresponding elements are identified in the figure. We can see that characteristic lines of Zn, Cu, Al, and Fe are all clearly visible in the spectra. Among them, we can find the characteristic lines of Zn I 468/472/481 nm (3d^10^4s5s ^3^S_1_–3d^10^4s4p ^3^P^0^_0_, 3d^10^4s5s ^3^S_1_–3d^10^4s4p ^3^P^0^_1_, 3d^10^4s5s ^3^S_1_–3d^10^4s4p ^3^P^0^_2_), Cu I 510/515/521 nm (3d^10^4p ^2^P^0^_3/2_–3d^9^4s^2 2^D_5/2_, 3d^10^4d ^2^D_3/2_–3d^10^4p ^2^P^0^_1/2_, 3d^10^4d ^2^D_5/2_–3d^10^4p ^2^P^0^_3/2_), Al I 394/396 nm (3s^2^4s ^2^S_1/2_–3s^2^3p ^2^P^0^_1/2_, 3s^2^4s ^2^S_1/2_–3s^2^3p 2P^0^_3/2_), and there are so many Fe lines that were not listed one by one. We can also find strong AlO molecular band (B^2^Σ^+^–X^2^Σ^+^) in the wavelength range from 460 to 530 nm as shown in Figure 4c. The generation of large number of oxygen atoms in plasma due to the effective dissociation of water molecules and the high oxidation rate of the Al target material result in the formation of AlO species [33]. By comparing the spectra measured in shallow sea and in laboratory, we can see the difference of the peak intensity on the observed characteristic lines of Zn, Cu, Al, and Fe. The difference on the peak intensity of the characteristic lines between the shallow-sea test and laboratory test may be due to the slight change on the lens-to-sample distance (LTSD). Another reason is that for the field test, the LIBS signal may be also influenced by the variation of oceanic parameters such as the pressure [17], temperature [34] and salinity [35]. It can be seen from the results of the comparison that there is almost no difference in other aspects of the characteristic lines (such as the width and wavelength positions of the characteristic peak) except for the intensity of the characteristic peak. This indicates the robustness performance of the LIBSea II system when working in the field shallow-sea environment.

**Figure 5:**
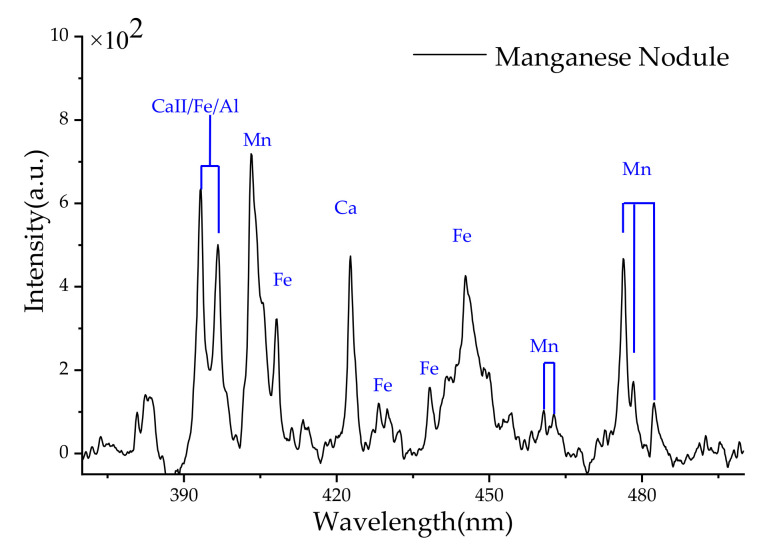
Typical spectra of a manganese nodule sample from deep sea in Jiaozhou Bay.

Furthermore, the nearshore detections of submerged mineral samples were performed. The measurements were made using 6 mJ single pulse energy, 400 ns observation gate delay, and 1 ms gate width, which were consistent with those of metal targets. Figure 5 shows the typical spectrum of a manganese nodule sample from deep sea after background subtraction processing, the characteristic lines of major metallic elements of Fe and Mn can be observed clearly. For the wavelengths overlap of Al at 394 and 396 nm with strong emissions of Ca (II) and Fe, they can’t be distinguished due to the limited resolution of the spectrometer. 

**Figure 6:**
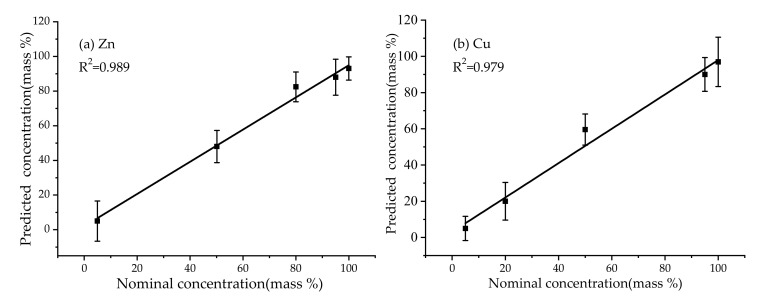
The correlation relationship between predicted concentration and nominal concentration of Zn (**a**) and Cu (**b**) based on multiple linear regression.

In order to verify the feasibility of LIBSea II for quantitative analysis of submerged solids, field experiments were also carried out in Jiaozhou Bay by using the Zn-Cu alloy samples with four different Zn/Cu mass ratios and two pure metal samples. The concentrations of Zn and Cu are ranging from 4.97% to 99.99% (S1: Zn 4.97% and Cu95.03%, S2: Zn 50.15% and Cu 49.85%, S3: Zn 80.01% and Cu 19.99%, S4: Zn 95.03% and Cu 4.97%, S5: Zn 99.99%, S6: Cu 99.99%). The samples were fixed on a one-dimensional linear rotating stage to avoid continuous breakdown in one site on the sample surface. Each spectrum was recorded with a delay time of 400 ns and an exposure time of 1 ms, and 20 replicate spectra were acquired for each sample. The original spectral data were pre-processed by background subtraction and then normalized by the total intensity of the whole spectrum. For quantitative analysis, multiple linear regression was used to build the calibration model in order to reduce the matrix effect and the instability of LIBS signals. The concentration of the element to be measured in the multiple linear regression model is expressed as ${Y=b}_{0}+\overset{n}{\underset{i=1}{\sum }}a_{i}X_{i}+\varepsilon_{i}$, where Y is the concentration of the element, X_i_ is the intensity of the selected analyte lines, ε_i_ is the error term, a_i_ is the regression coefficient of multiple linear regression and b_0_ is the linear regression constant. The intensities of three Zn lines (Zn I 468 nm, Zn I 472 nm, Zn I 481 nm) and three Cu lines (Cu I 510 nm, Cu I 515 nm, Cu I 521 nm) were selected as the input variables for the multiple linear regression model. The correlation relationships between the predicted concentration and reference concentration of Zn and Cu are shown in Figure 6. We can see a relatively high linearity of the calibration curve with the correlation coefficients (R^2^) of 0.989 and 0.979 for Zn and Cu, respectively. These results indicate that LIBSea II for in-situ direct detection and quantitative analysis of submerged solids in real seawater environment. More works are still needed to test the analytical performance of the system, as well as the influence of oceanic environments on in-situ quantitative analysis of submerged solid targets.

### 3.3. Preliminary Results in sea Trials

**Figure 7:**
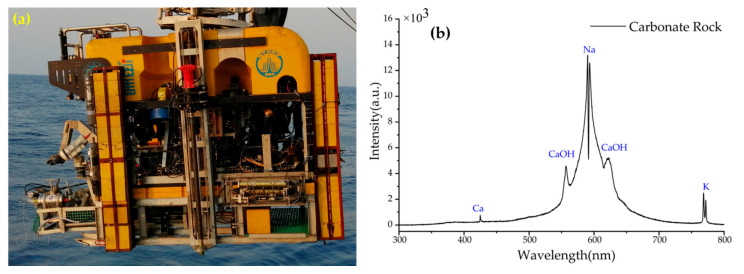
(**a**) LIBSea II mounted on the Haima ROV for in-situ experiments in the Haima cold seep area (**b**) Typical spectra of carbonate rock measured in deep sea.

A deep-sea trial was carried out at the Haima cold seep area of the South China Sea in May 2019. The main purpose of the trial is to verify that in-situ experiments can be performed in deep sea.Figure 7a shows the picture of LIBSea II system deployed on the Haima ROV. During the sea trial, as ROV rising to sea surface and dived to seafloor, we obtained a large number of depth profiles data which were not shown, since it was not the focus of this paper. A carbonate rock sample, which was typical in cold seep area, was used for deep-sea LIBS detection. The spectrum of the carbonate rock sample was successfully obtained at the depth of 1400 m, as shown in Figure 7b. From the spectrum in Figure 7b, we can see clearly the atomic line of Ca I 422 nm, Na I 588/589 nm, and K I 766/769 nm. It is worth noting that the strong molecular bands of calcium hydroxide are also detectable on either side of the Na I 588/589 nm line. The band around 554 nm is assigned to the green system B^2^Σ^+^-X^2^Σ^+^ of the triatomic radical CaOH, while the band around 623 nm is assigned to the orange-red system A^2^Π-X^2^Σ^+^ of CaOH [36,37]. The previous research revealed that CaOH molecular emissions (homogeneous distribution, longer lifetime, and high emission intensity) can improve the analytical performances of underwater LIBS by using the CaOH molecular bands instead of Ca II and Ca I lines. And the quantification results of CaOH are excellent with higher stability, less self-absorption, and reduced matrix effect. The acquisition of CaOH molecular signal may provide a new approach for improving the performances of underwater LIBS analysis [38]. The results showed that the LIBSea II system was feasible in deep-sea submerged solid detection. In the future works, we plan to integrate an auto-focus module in LIBSea II system and develop the signal processing methods for qualitative analysis, aiming to enable LIBS technology to be practically applied for the in-situ exploration in deep-sea polymetallic areas.

## 4. Conclusions

In this work, a more compact and lighter underwater LIBS system named LIBSea II was developed for the in-situ detection of submerged solid samples. The system can be deployed with ROVs and is capable of performing in-situ, multi-element analysis of submerged solid at depths of up to 5000 m. The system is encapsulated in a pressure housing with the size of Φ 190 mm × L 588 mm. The system consists of a Nd:YAG laser at 1064 nm, an optical spectrometer, optical layout, a power supply module and an internal environment sensor. The laser energy of 6 mJ and delay time of 400 ns were firstly determined by optimizing the system experimental parameters in the laboratory. Field tests of the system in shallow sea were then performed with various pure metal, alloy samples and manganese nodule samples from deep sea to show the detection performance of the LIBSea II system. There was a good consistency between the spectra measured in shallow sea and in laboratory, and the feasibility of LIBSea II for direct detection and quantitative analysis of submerged solids were also demonstrated. The deep-sea trial was carried out in the South China Sea in May 2019 by deploying on the Haima ROV to verify that the system can be operated in deep sea. The atomic lines of K, Na, Ca and strong molecular bands of CaOH from a carbonate rock sample were obtained for the first time at the depth of 1400 m, which proved the feasibility of LIBSea II in deep-sea environment. Our future work will focus on how to apply this equipment to in-situ quantitative and qualitative analysis of submerged sediments and rocks. We plan to place the system on a high-precision motor and use ROV’s manipulator for precise focusing of the system. In addition, the adoption of more advanced data processing methods will be helpful in in-situ quantitative and qualitative analysis of submerged sediments and rocks. Furthermore, the combination of extensive laboratory data and sea-trials data to establish the correction model between LIBS signals and environment factors seems particularly important. It is hoped that the underwater LIBS system through optimization and upgrading can make important contributions to the deep-sea explorations.

## Figures and Tables

**Table 1:** General Specifications of the LIBSea II System.

Module	Apparatus	Specifications
Mechanics	Pressure vessel	7075 Aluminum alloy, anodized surface 588 × 190/length (mm) × diameter (mm) 28 kg (12 kg)/weight in air(water) 50 MPa/pressure proof 62.5 MPa/hydrostatic pressure
Optics	Laser: (Montfort, M-NANO)	1064 nm, Nd:YAG ≤40 mJ/pulse energy 2–25 Hz/adjustable repetition rate 10 ns/pulse width 258 × 78 × 64/dimension (mm^3^)
	Spectrometer: (Avantes, AvaSpec-Mini2048)	260–800 nm/wavelength range 0.63 nm/spectral resolution 1280 ns/minimum detection delay 1 ms/minimum integration time 120 × 90 × 35/dimension (mm^3^)
	Optical window	Fused silica 19 × 16.35/diameter (mm) × thickness (mm) 15 mm/focal length 50 MPa /pressure proof 62.5 MPa/hydrostatic pressure
	Layout	Confocal collection
Electronics	Power supply	110 V AC/48 V DC from ROV
	Delay generator	800–4400 ns/Q-Switch out from laser
	Microcomputer	PC 104/Advantech PCM-3363
	Communication	Ethernet/USB/RS232/RS485 Remote desktop connection
